# Influence of acupuncture intensity on analgesic effects in AA rat models

**DOI:** 10.3389/fbioe.2024.1502535

**Published:** 2024-12-11

**Authors:** Yi-Xuan Wang, Yu-Hang Liu, Zi-Liang Zhang, Xuan Qiao, Ying-Chen Li, Liu-Jie Ren, Guang-Hong Ding, Wei Yao, Yi Yu

**Affiliations:** ^1^ Department of Aeronautics and Astronautics, Fudan University, Shanghai, China; ^2^ Shanghai Key Laboratory of Acupuncture Mechanism and Acupoint Function, Shanghai, China; ^3^ Eye and ENT Hospital of Fudan University, Shanghai, China; ^4^ College of Medical Instruments, Shanghai University of Medicine and Health Sciences, Shanghai, China

**Keywords:** acupuncture intensity, analgesia, mast cell, adjuvant arthritis, robot arm

## Abstract

**Objective:**

To investigate the influence of acupuncture lifting-thrusting frequency and amplitude on the analgesic effects, and its correlation with mast cell degranulation.

**Methods:**

Acute adjuvant arthritis (AA) rat models were employed. Robot-arm aided lifting-thrusting acupuncture therapy was conducted with various frequencies (ranging from 0.5 to 4 Hz) and amplitudes (ranging from 0.5 to 2.0 mm). The rats’ pain thresholds were measured multiple times before and after the therapy, and the analgesic effects were evaluated using the pain threshold recovery rate (PTRR), a normalized index. The mast cell degranulation rate (MCdR) at the acupoint was calculated, and a correlation analysis between PTRR and MCdR was performed.

**Results:**

Acupuncture therapy partially restored the pain threshold affected by arthritis. The analgesic effects were influenced by stimulus frequency and amplitude, with best outcomes occurring at an intermediate optimal frequency of 1.0 Hz and amplitude of 1.0–1.5 mm. Similarly, the MCdR peaked at the optimal frequency and amplitude.

**Conclusion:**

Our animal experiment suggests that optimal analgesic effects can be achieved with stimulation at an optimal intensity. This intensity-effect correlation appears to originate from mast cell activation rates under different mechanical stimulus.

## 1 Introduction

As a traditional minimally invasive treatment in Chinese medicine, acupuncture has been extensively utilized in clinical practice, particularly in pain management (Wu et al., 2016; Zhou et al., 2022). Acupuncture treatment involves partly inserting a tiny needle into the acupoint, and then exerting external force by mechanically manipulating the needle handle, such as lifting-thrusting or rotating. The efficacy of acupuncture is closely related to the manipulating parameters, such as duration, direction, and intensity (Shi et al., 2012; Lyu et al., 2019). However, these parameters heavily depend on the experience and preferences of the practitioner, leading to significant variability in therapeutic outcomes. Moreover, the relationship between acupuncture parameters and therapeutic efficacy remains unclear, and the underlying mechanisms are largely unexplored.

Difference in the manipulating intensities may lead to varying therapeutic effects (Hong et al., 2017). Several studies highlight the significance of acupuncture frequency. Song et al. found that manual rotating acupuncture (4 Hz) had superior analgesic effects compared to 2 Hz in acute adjuvant arthritis (AA) rat models (Song et al., 2021). Liu et al. observed that treatment with four different frequencies of manual acupuncture (1 Hz, 2 Hz, 3 Hz, and 4 Hz) affected the gastric motility amplitude to varying degrees (Liu et al., 2019). Armour et al. reported that high-frequency manual acupuncture was more effective than low-frequency acupuncture in reducing the intensity and duration of menstrual pain (Armour et al., 2017). However, the reason for the observed frequency dependence remains unexplained, partly because the underlying mechanism of acupuncture itself is still not fully understood and is debated (Nurwati et al., 2020).

Attempts to uncover the mystery of acupuncture focus on various physiological indices spanning different scales and organs. Most researchers focus on the local and central nervous system, such as the release of endorphins (Harbach et al., 2007) caused by acupuncture, which may further regulate physiological changes (such as heart rate) in the body (Luo et al., 2019). Some researchers are interested in the local mechanical and biological responses of the stimulated acupoint, including the activation of mast cells (Luo et al., 2013), which is one of the most important findings (Bae et al., 2021). It is repeatedly reported that mast cells are mechanically activated (Li et al., 2022), undergo degranulation, and release a series of biochemical substances (Mukai et al., 2018), including serotonin (Li et al., 2023; Dimitrov et al., 2017), histamine (Ding et al., 2018; Wang et al., 2022), leukotrienes, and many others. These released substances may play a positive role in analgesia. Animal experiments have shown that the analgesic effect of acupuncture is related to mast cell degranulation, and the released histamine (a biomarker of inflammation and allergic reactions) is involved in both analgesic and immune responses (Huang et al., 2018).

In this study, AA rat models were employed to investigate the influence of acupuncture parameters on analgesic effects. Robot-arm aided lifting-thrusting acupuncture therapy was conducted with various frequencies (ranging from 0.5 to 4 Hz) and amplitudes (ranging from 0.5 to 2.0 mm) to accurately control stimulus parameters. The pain thresholds of the animals were measured at multiple time points before and after the therapy to evaluate the therapeutic outcomes. Moreover, mast cell degranulation was assessed to reveal its intrinsic role in the underlying mechanism.

## 2 Materials and methods

### 2.1 Animals

This study utilized seventy-eight healthy adult male Sprague Dawley (SD) rats (SPF grade, weighted 200 ± 20 g). The rats were housed in the Animal Experiment Center of Shanghai University of Traditional Chinese Medicine under controlled conditions (22°C–25°C, 50%–60% humidity, 12-hour light/dark cycle). All animal experiments were conducted in accordance with the ethical guidelines and were approved by the local Animal Ethics Committee (Approval No: PZSHUTCM2308010011).

The animals were randomly divided into the following groups: the robot-arm acupuncture group (RA, further subdivided into 8 subgroups), the manual acupuncture group (MA), the Model group (Model), the needle-retaining group (NR), the clemastine fumarate (a long-acting antihistamine) combined with acupuncture group (Cle + Acu), and the saline combined with acupuncture group (Saline + Acu). Each group/subgroup consisted of six rats.

### 2.2 Acute adjuvant arthritis model (AA model)

**FIGURE 1:**
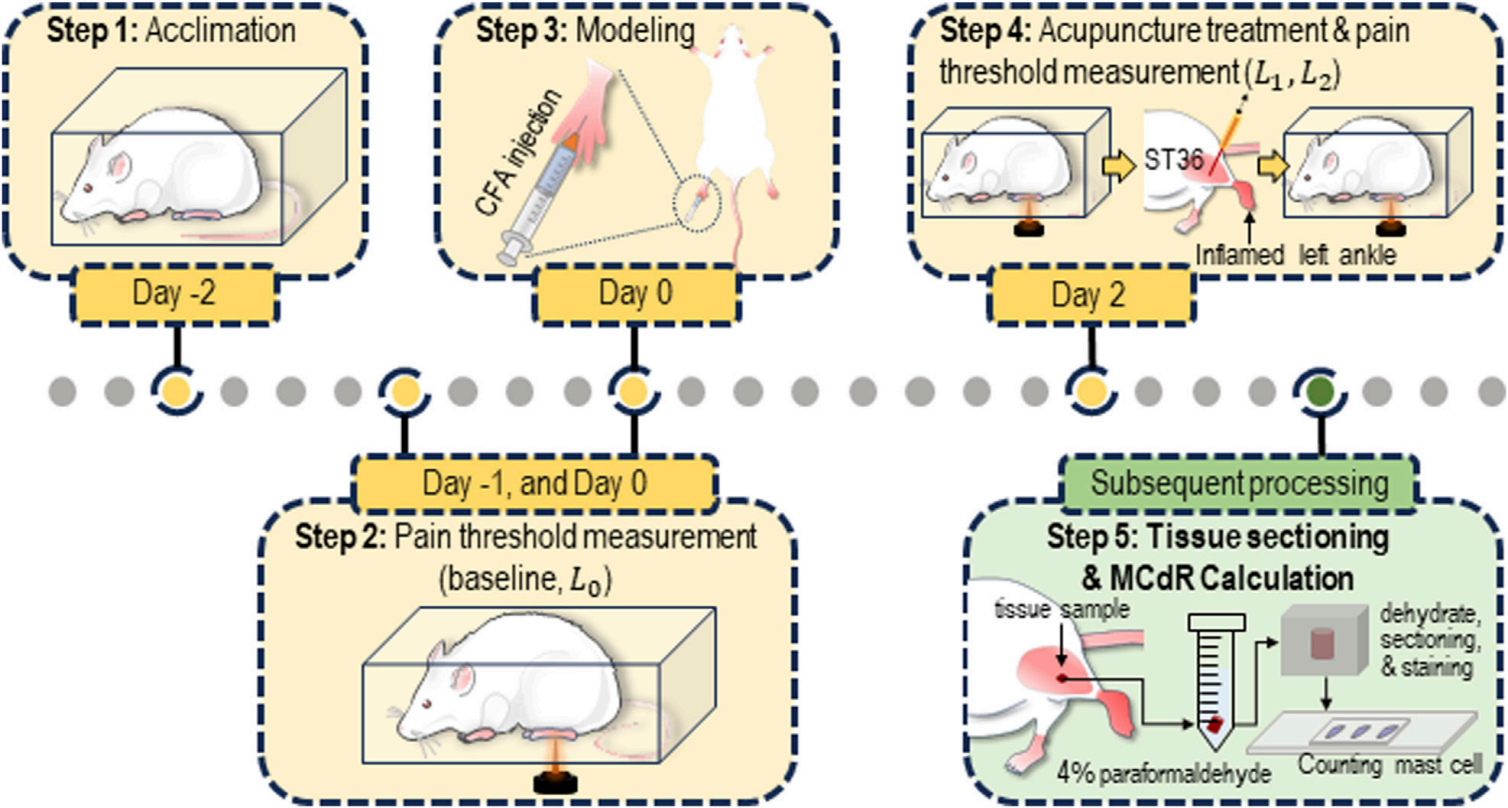
Protocol for the animal experiment. The rats were acclimated for 3 days (from Day −2 to Day 0) prior to the establishment of the acute adjuvant arthritis (AA) model. Acupuncture was performed on Day 2. Pain threshold measurements were conducted on Day −1, Day 0 (before modeling), Day 2 (before and after the acupuncture treatment). After the measurement, tissue samples from the ST36 acupoint were collected and sectioned for estimation of the mast cell degranulation rate (MCdR).

The current study adopted the widely used AA rat model for acupuncture research (Li et al., 2023). The model was induced by injecting complete Freund’s adjuvant (CFA, 200 μL) into the left ankle joint cavity of the animal (see Figure 1). Local ankle swelling, characterized by noticeable redness and increased volume, was observed 48 h after CFA injection. Simultaneously, clear behavioral disability (altered gait and reduced activity) occurred.

### 2.3 Behavioral tests of pain threshold

Thermal pain threshold measurement was adopted to assess the pain sensitivity of the animals. The animals were acclimated to the testing cage for approximately 30 min daily over 3 days before the experiment to ensure they were habituated to the environment. During the test, a radiant heat beam (IITC336G, IITC Life Science, United States) was directed at the left paw of the animal, and the latency time (in seconds) before the animal retracted its paw was measured to estimate the thermal pain threshold, defined as paw withdrawal latency (PWL) (Shen et al., 2021). To prevent potential overheating damage, the maximum heating time was limited to 20 s. The tests were repeated three times with 10-min intervals, and the median value was used as the pain threshold.

Each animal underwent four behavioral tests at different time points throughout the experiment (see Figure 1). Two tests were conducted during the acclimation period (Day −1 and Day 0, with the average of the two results recorded as $L_{0}$ ), one test after the AA model was established (Day 2, with the result denoted as $L_{1}$ ), and the final test after the treatment (Day 2, with the result denoted as $L_{2}).$ The value $L_{0}$ defined the baseline pain threshold for each animal, which varied individually. To eliminate the difference, a dimensionless index called the pain threshold recovery ratio (PTRR, see Equation 1) was calculated as:

$$PTRR=\frac{{L_{2}-L}_{1}}{L_{0}-L_{1}}$$ (1)

where $L_{0}-L_{1}$ is the drop in pain threshold after the model establishment, indicating increased sensitivity to heat stimuli, and $L_{2}-L_{1}$ estimates the rise in pain threshold after treatment. The ratio of the rise to the drop defines the dimensionless PTRR, which evaluates the analgesic effect. Briefly, when PTRR≈0 , the treatment has no effect on the pain threshold; when PTRR→1 , the pain threshold returns to baseline, indicating the treatment’s effectiveness in analgesia. Given that the analgesic effects may dissipate over time, $L_{2}$ was measured within 30 min after treatment for all animals.

### 2.4 Treatment interventions

**FIGURE 2:**
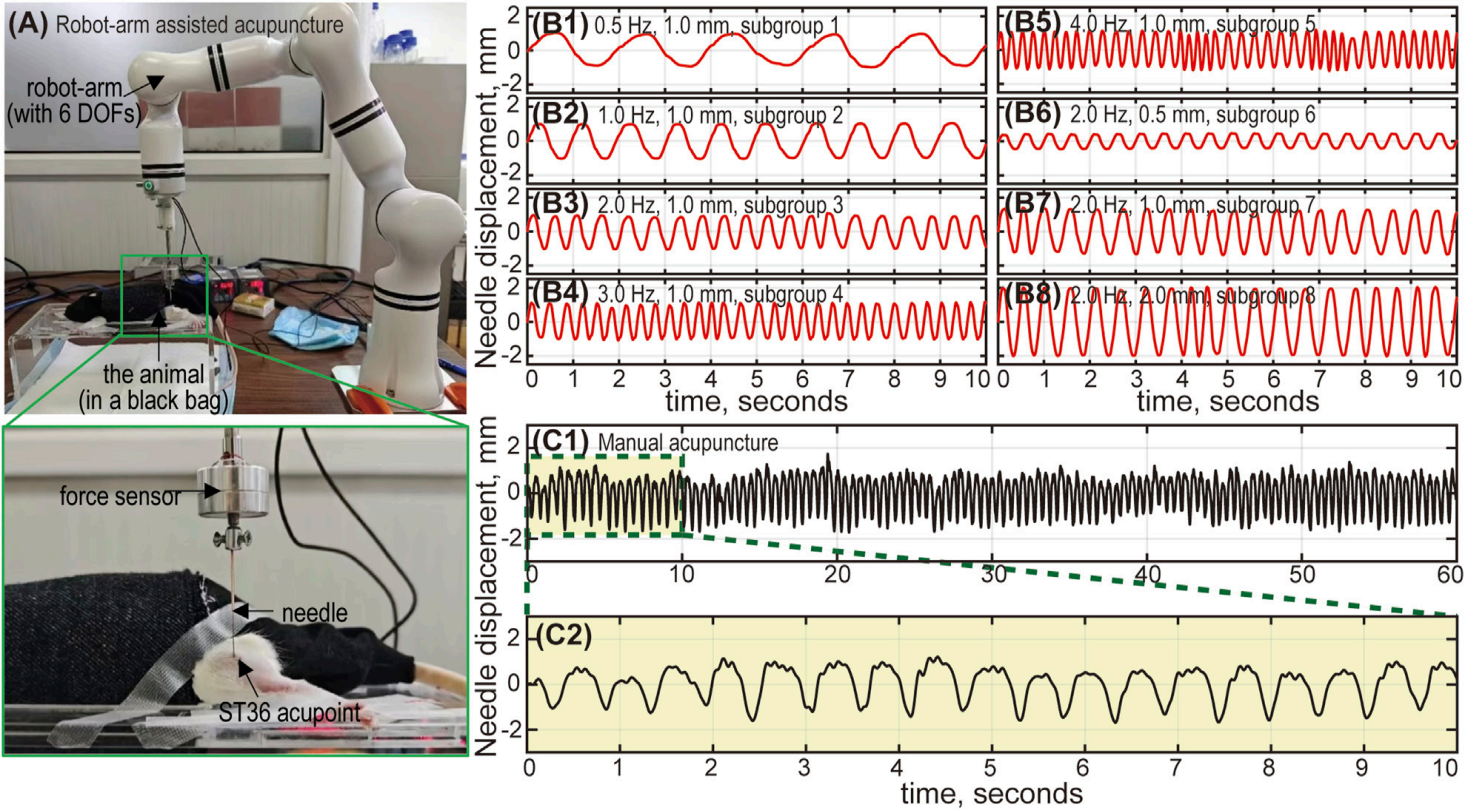
The robot-arm assisted acupuncture system. **(A)** The animal is placed in a black bag to relieve stress. The needle is connected to a 6-DOF (degrees of freedom) robot arm, and inserted into the ST36 acupoint (left foot) of the animal. The robot-arm moves up and down, mimicking the lifting-thrusting acupuncture technique, with frequency and amplitude controlled by an in-house Python program. A force sensor between the needle and the robot-arm monitors the applied force. Needle movement during robot-arm assisted and manual acupuncture: Subfigures (B1-B8) show the needle displacement corresponding to subgroups 1 to 8. The actual needle movement under robot-arm control generally aligns with our designed parameters (frequency and amplitude). Errors are relatively larger with high frequency and large amplitude combinations (subgroup 4, 5 and 8), but remain within 5%. Subfigure (C1) shows the needle movement for manual acupuncture, lasting 60 s. Subfigure (C2) provides an enlarged view of the displacement curve (10 s). The amplitude is approximately 1 mm, and the frequency is around 2 Hz.

To investigate the influence of acupuncture intensity on analgesic effects, multiple treatment interventions were applied to the aforementioned animal groups (see Table 1). Animals in the RA groups received acupuncture at the ST36 acupoint using a robot-arm (RM65, Realman, China) to precisely control various combinations of stimulus parameters for each subgroup (see Figure 2A). The MA group received manual lift-thrusting acupuncture (frequency ∼2.0 Hz, amplitude ∼1.0 mm). No interventions were applied for the Model group. In the NR group, an acupuncture needle was inserted at the acupoint with no further manipulations. In the Cle + Acu group, an intramuscular injection of clemastine fumarate (200 μL) was administered into the ST36 acupoint 30 min prior to acupuncture treatment (using robot-arm, frequency 1.0 Hz, amplitude 1.0 mm). Similarly, the Saline + Acu group received an injection of Saline (200 μL) before the robot-arm assisted acupuncture.

**TABLE 1:** An overview of treatment interventions of different animal groups.

Group	Interventions	Subgroups	Stimulus parameters	
			Frequency, Hz	Amplitude, mm
RA	Robot-arm assisted acupuncture treatment (ST36, 10 min)	1*	0.5	1.0
		2*	1.0	1.0
		3*^,+^	2.0	1.0
		4*	3.0	1.0
		5*	4.0	1.0
		6+	2.0	0.5
		7+	2.0	1.5
		8+	2.0	2.0
MA	Manual acupuncture treatment	—	∼2.0	∼1.0
Model	No intervention	—	—	—
NR	Inserting the needle, without stimulus, for 10 min	—	—	—
Ale + Acu	Clemastine fumarate pre-injection before acupuncture	—	2.0	1.0
Saline + Acu	Pre-injection of saline before acupuncture	—	2.0	1.0

* These subgroups were adopted to analyze the influence of acupuncture frequency.

+ These subgroups were adopted to analyze the influence of acupuncture amplitude.

Both manual and robot-arm assisted acupuncture were performed at the ST36 point (approximately 5 mm below the fibula head, on the lateral side behind the knee joint) (Oh and Kim, 2022). Fine and sharp steel needles (diameter 0.3 mm, length 25 mm, Huatuo, China) were used for acupuncture stimulus. All acupuncture treatments (including the NR group) lasted 10 min.

For robot-arm assisted acupuncture (RA group), the needle moved sinusoidally up and down, mimicking the common lifting-thrusting acupuncture technique. Custom Python scripts were used to precisely control the stimulus frequency (0.5–4.0 Hz) and amplitude (0.5–2.0 mm). The frequency range used in this study was consistent with clinical settings to ensure relevance to human acupuncture practices. However, the amplitude was adjusted to suit animal studies, taking into account the smaller size and anatomical characteristics of the animal models. This adjustment ensured effective stimulation while maintaining biological feasibility.

### 2.5 Verification of robot-arm assisted acupuncture

To verify the precise control of the robot-arm, a Laser Doppler vibrometer (CLV-2500, PolyTec, Germany) was used to measure the displacement of the needle movement driven by the robot arm. The results demonstrated that both the frequency and amplitude could be accurately controlled, with a relative error below 5% (for both frequency and amplitude) (see Figure 2B). Additionally, the needle displacement under manual control was also measured to provide a quantitative estimate of the stimulus frequency and amplitude for the MA group (see Figure 2C).

### 2.6 Estimation of mast cell degranulation rate (MCdR)

After the behavioral tests, the animals were euthanized by CO_2_ inhalation, in accordance with the American Veterinary Medical Association (AVMA) Guidelines. Each animal was placed individually into a 10 L euthanasia chamber, and CO_2_ gas was gradually introduced at a flow rate of 10%–30% of the chamber volume per minute, ensuring a gradual increase in gas concentration to minimize distress.

Following euthanasia, tissue samples from the ST36 acupoint were immediately collected and fixed in 4% paraformaldehyde. The tissues were then dehydrated, embedded in paraffin, and sectioned into thin slices (5 μm). The sections were stained with toluidine blue, which allowed mast cells to be easily distinguished under an optical microscope (NTB900-FL, Ningbo Yongxin Optical, China). Un-degranulated mast cells appeared as circular, oval, or spindle-shaped isolated dots, while degranulated cells exhibited dispersed purple granules around the cell body (see Supplementary Figure S1 in the Supplementary Material). To calculate the MCdR, six sections for each animal were randomly sampled (three sections containing muscle tissue and three containing skin). The total number of degranulated and un-degranulated mast cells were counted, and the MCdR was calculated as the ratio of degranulated cells to total mast cells.

### 2.7 Statistical analysis

All pain threshold results and MCdR values were analyzed using the Statistics and Machine Learning Toolbox in Matlab (R2023a, MathWorks, United States). Data were expressed as mean ± standard deviation. Group comparisons were conducted using one-way analysis of variance (ANOVA), followed by Tukey’s post-hoc tests for pairwise comparison. A P-value <0.05 was considered statistically significant.

## 3 Results

### 3.1 Manual and robot-arm assisted acupuncture

**FIGURE 3:**
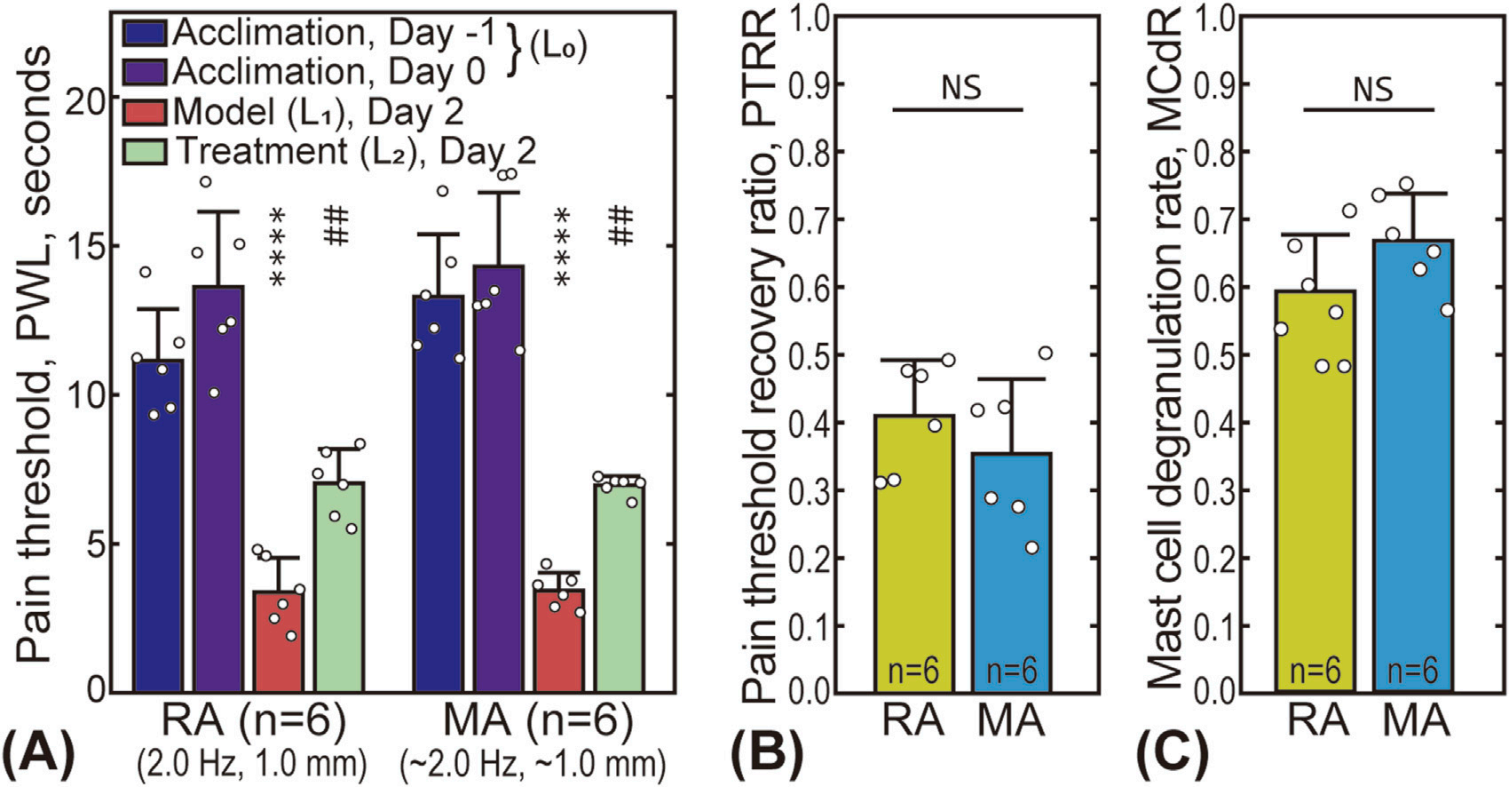
Comparison between robot-arm assisted acupuncture (RA group, subgroup 3) and manual acupuncture (MA group). **(A)** The pain thresholds of the animals in the RA and MA groups at four time points. The star “*” markers indicate the statistical difference between the pain threshold baseline and that after model establishment ( $L_{0}$ and $L_{1}$ ), **** P<0.0001 . The sharp “#” markers indicate statistical difference between pain threshold before ( $L_{1}$ ) and after ( $L_{2}$ ) the treatment, ## P<0.01 . **(B, C)** present the statistical results of the PTRR and MCdR, respectively. “NS” indicates no significant difference ( P>0.05 ) between the two groups.

The effectiveness of using the robot-arm for acupuncture treatment was validated by comparing the MA and RA groups. Subgroup 3 of the RA group was selected due to its similar stimulus parameters to the MA group (stimulus frequency 2.0 Hz, amplitude 1.0 mm). Figure 3A presents the pain threshold of the animals at the four time points. For both groups, the PWL significantly decreased after the AA model was established (from 10 to 18 s to less than 5 s), and partially recovered (to 6–8 s) after the treatment, indicating that both manual and robot-arm assisted acupuncture are effective in alleviating pain. A comparison of the PTRR between the two groups is shown in Figure 3B, with a value of 0.41 ± 0.08 for the RA subgroup and 0.35 ± 0.11 for the MA group, with no statistically significant difference. Figure 3C shows that the MCdR for the two groups are also similar (0.59 ± 0.08 for the RA subgroup and 0.67 ± 0.07 for the MA group).

### 3.2 Influence of stimulus intensity on analgesia

**FIGURE 4:**
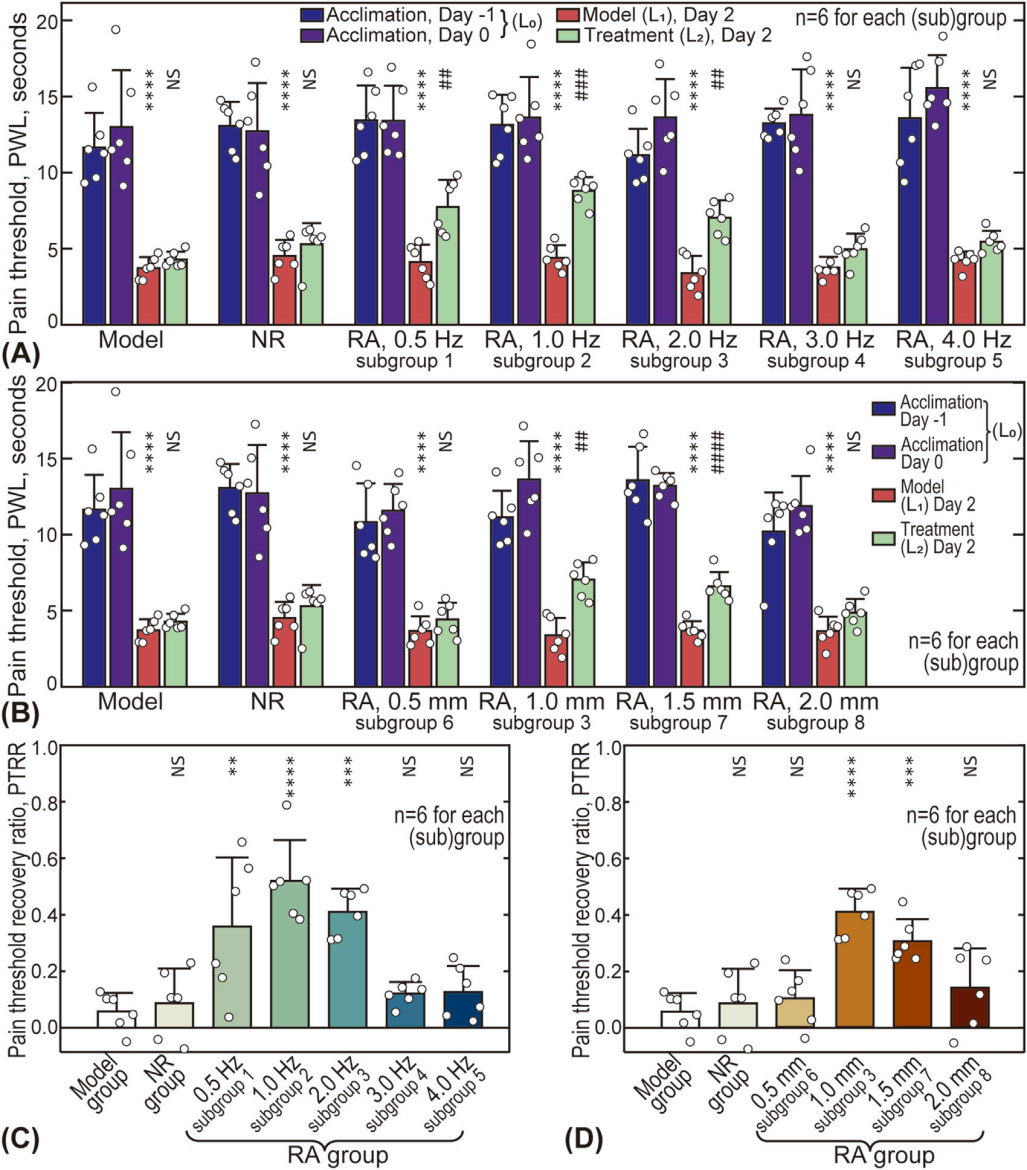
Influence of acupuncture intensity on analgesic effects. **(A)** Influence of acupuncture frequency on pain thresholds of animals; **(B)** Influence of acupuncture amplitude; **(C)** Influence of frequency on pain threshold recovery ratio (PTRR); **(D)** Influence of amplitude on PTRR. In subplot A and B, differences between $L_{1}$ and $L_{0}$ , and $L_{2}$ and $L_{1}$ were analyzed. In subplot **(C, D)**, the PTRR values of other groups were pairwise compared with the Model group. NS: statistically not significant, P>0.05 ; * or #: P<0.05 ; ** or ##: P<0.01 ; *** or ###: P<0.001 ; **** or ####: P<0.0001 .

The effects of acupuncture intensity (represented by stimulus frequency and amplitude) on analgesic outcomes are shown in Figure 4, with the original data available in the Supplementary Material (Supplementary Table S1). Five different frequencies (0.5, 1.0, 2.0, 3.0, and 4.0 Hz) were tested and compared with the Model and NR groups (see Figures 4A, C). For all groups/subgroups, the modeled pain threshold $L_{1}$ was significantly decreased compared with the baseline $L_{0}$ , but the thresholds after treatment $L_{2}$ varied across groups. For the Model and NR groups, no recovery was observed, whereas significant recovery was seen at 0.5, 1.0, and 2.0 Hz. The PTRR values (Figure 4C) suggest that the PTRR is optimal (0.52 ± 0.14) after 1.0 Hz acupuncture treatment.

The influence of amplitude is shown in Figures 4B, D, with four different amplitude values (0.5, 1.0, 1.5, and 2.0 mm) compared. Similarly, the pain threshold after treatment $L_{2}$ depended on the amplitude, with optimal recovery observed at 1.0–1.5 mm (0.41 ± 0.08 for 1.0 mm, and 0.30 ± 0.08 for 1.5 mm, see Figure 4D). For lower (0.5 mm) or higher (2.0 mm) amplitudes, the pain threshold recovery was not significant.

### 3.3 Influence of stimulus intensity on mast cell degranulation

**FIGURE 5:**
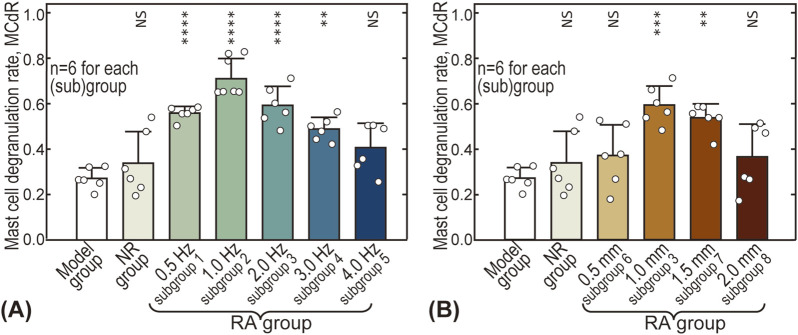
Influence of acupuncture intensity on mast cell degranulation rate (MCdR). **(A)** Influence of acupuncture frequency; **(B)** Influence of acupuncture amplitude. The MCdR values of other groups were pairwise compared with the Model group. NS: statistically not significant, P>0.05 ; *: P<0.05 ; **: P<0.01 ; ***: P<0.001 ; ****: P<0.0001 .

Figure 5 gives the influence of acupuncture intensity on MCdR at the ST36 acupoint. The baseline MCdR with no stimulus applied (Model group) is 0.27 ± 0.05, indicating that approximately one-fourth of the mast cells were activated at rest. The MCdR is also dependent on acupuncture frequency, reaching a maximum (0.71 ± 0.09) around 1.0 Hz (see Figure 5A). Optimal acupuncture amplitude is also observed around 1.0–1.5 mm, where the MCdR reaches 0.59 ± 0.08 and 0.54 ± 0.06, respectively.

### 3.4 Effect of antihistamine

**FIGURE 6:**
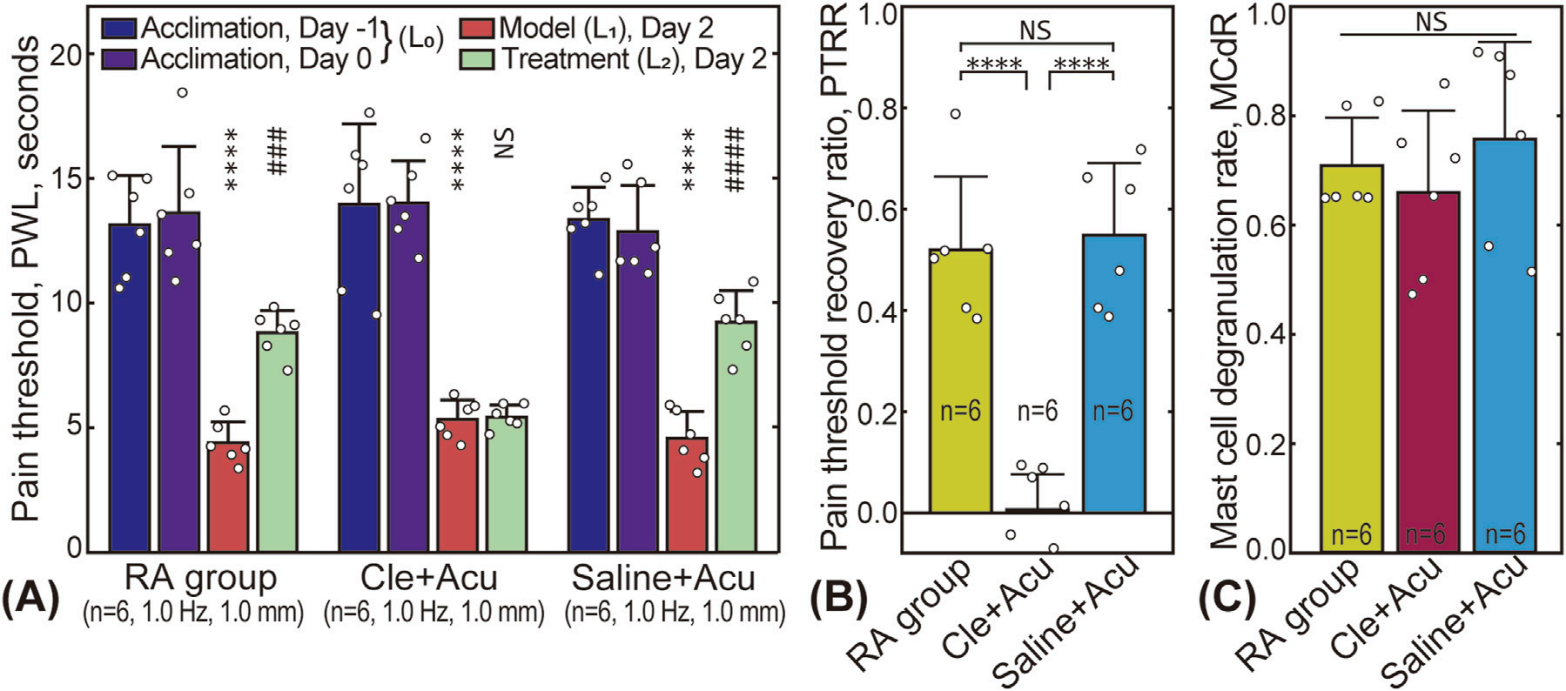
Influence of clemastine fumarate injection. **(A)** Pain threshold results; **(B)** PTRR results for the RA (subgroup 2), Cle + Acu, and Saline + Acu groups; **(C)** Corresponding MCdR results. NS: statistically not significant, P>0.05 ; * or #: P<0.05 ; ** or ##: P<0.01 ; *** or ###: P<0.001 ; **** or ####: P<0.0001 .

The effect of clemastine fumarate (antihistamine) on PTRR and MCdR is shown in Figure 6. As shown in Figures 6A, B, injecting saline into the acupoint before the treatment did not affect pain relief. In contrast, injection of clemastine fumarate blocked the therapeutic effect, and the PTRR of the Cle + Acu group was close to 0. No significant difference in MCdR was found among the three groups.

### 3.5 Correlation between PTRR and MCdR

**FIGURE 7:**
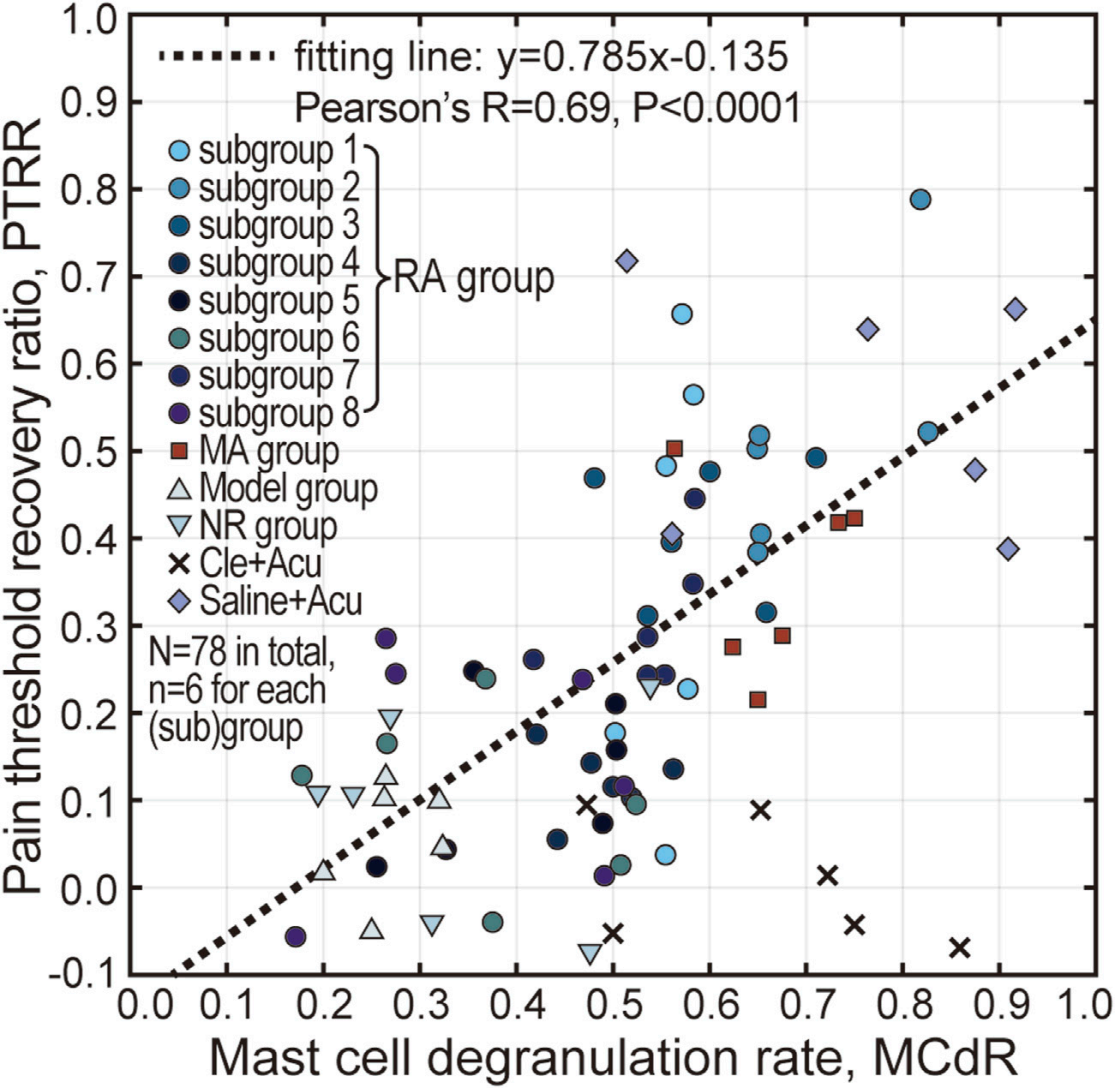
Correlation analysis between PTRR and MCdR. Pearson’s correlation analysis was conducted, indicating a strong positive correlation between PTRR and MCdR, except for the Cle + Acu group. The dotted line represents the linear fit of the data points.

A correlation analysis between PTRR and MCdR was conducted, and all animal data (N = 78) from this study are presented in Figure 7. The PTRR and MCdR were strongly positively correlated (with the exception of the Cle + Acu group, where antihistamine had been injected), yielding a Pearson’s r of 0.69, and a very small P-value (note that data from the Cle + Acu groups were not included in the correlation analysis). These results suggest an intrinsic relationship between acupuncture analgesia and mast cell degranulation.

## 4 Discussions

### 4.1 Influence of acupuncture parameters on analgesic effects

Understanding the relationship between acupuncture parameters (such as duration, intensity, manipulating techniques, and acupoint selection) and therapeutic outcomes is crucial for clinical practice, and has thus attracted significant interest from researchers (Wu et al., 2024). However, there is currently a lack of biomechanical research focusing on the impact of quantitative mechanical parameters (e.g., frequency, amplitude). In this study, the effects of acupuncture lifting-thrusting intensity on analgesia were systematically investigated using AA rat models. We found that there are optimal frequency (∼1.0 Hz) and optimal amplitude (1.0–1.5 mm) values for acupuncture stimulation to achieve maximal analgesic effects (see Figure 4). Moreover, the influence of acupuncture intensity, particularly the optimal frequency and amplitude, was reflected in the MCdR results (see Figure 5).

The existence of optimal parameters is supported by several other works. Liu et al. reported that manual acupuncture at 2 Hz was most effective in improving gastric motility in rats with atropine-induced gastric hypomotility, while lower (1 Hz) and higher frequencies (3 Hz and 4 Hz) were less effective (Liu et al., 2019). Hong et al. demonstrated that lifting-thrusting needle manipulations at frequencies of 2 and 3 Hz were more effective in inhibiting acute visceral nociception in rats with gastric distension than 0.5 and 1 Hz (Hong et al., 2015). The influence of acupuncture amplitude has been less frequently reported, as most studies have adopted manual acupuncture, where it is difficult to precisely control the needle amplitude. Fortunately, this limitation is overcome with robot-arm assisted acupuncture, which is as effective as manual treatment (see Figure 3) (Yu et al., 2014; Turkistani et al., 2021; Zhi et al., 2019). The use of a robot-arm in this study not only reduces human labor, but also allows for precise control of acupuncture frequency and amplitude, ensuring both quantitative accuracy and long-term reliability.

Additionally, treatment duration plays an important role in analgesia. To minimize experimental time, this study employed a standard acupuncture duration of 10 min. However, extending the treatment duration to 20–30 min may lead to even better outcomes (see Supplementary Figure S2 in the Supplementary Material).

### 4.2 The role of mast cell degranulation

The significance of mast cell degranulation in initiating acupuncture effects at the local acupoint has been extensively reported (Bae et al., 2021; Wang et al., 2015). The correlation between PTRR and MCdR (see Figure 7) not only supports the existing literature, but also implies that the influence of acupuncture intensity is closely related to, or possibly caused by, difference in mast cell activation.

Histamine, a substance released during mast cell degranulation (Brzezińska-Błaszczyk et al., 2011), plays a role in acupuncture-induced analgesia. Histamine regulates neurotransmission and immune responses by binding to various histamine receptors, such as H1 and H2 receptors, thereby influencing pain perception and acupuncture-induced analgesia. Additionally, histamine modulates inflammatory responses and vascular permeability, which further impacts pain signal transmission. During acupuncture, histamine release may cause local vasodilation and tissue inflammation, directly affecting pain conduction pathways and reducing pain perception (Zhang et al., 2018). Our study shows that pre-injection of antihistamine substances can effectively block the analgesic effect. In the Cle + Acu group, despite significant mast cell degranulation (see Figure 6C), the analgesic effect was minimal (see Figure 6B). Similar results have been reported by Huang et al. (Huang et al., 2012).

However, the exact mechanism by which different acupuncture frequencies and amplitudes alter the mast cell degranulation remains unclear. One possible explanation lies in the bio-mechanical interactions between the needle and tissue during acupuncture. Langevin et al. reported that connective tissues are essential for effective needle-tissue coupling (Langevin et al., 2001; Langevin et al., 2002; Langevin and Yandow, 2002; Langevin et al., 2007). Some researchers propose that good needle-tissue coupling is necessary for transmitting external forces to the acupoint, inducing significant strain and stress within the tissue. This mechanical stimulus may activate local mast cells, ultimately leading to their degranulation (Li et al., 2022). Therefore, it is possible that needle-tissue interactions are sensitive to the acupuncture frequency and amplitude, as well as other factors such as mechanical properties of the acupoint, needle insertion depth, and manipulation techniques (e.g., needle lifting-thrusting or rotating).

### 4.3 Limitations and prospects

Although this study provides quantitative values for optimal frequency and amplitude, these data cannot be directly applied to clinical practice due to differences between animals and humans, as well as the variability introduced by factors such different diseases, symptoms, and acupoint selection. The primary contribution of this work is to confirm that the stimulus intensity plays a crucial role in acupuncture effectiveness, with mast cell degranulation being a key mediator. Future research is needed to better elucidate how acupuncture parameters are linked to local tissue mechanical responses (such as needle-tissue coupling), and the biomechanical reactions of mast cells.

## 5 Conclusion

This study demonstrates that acupuncture intensity (frequency and amplitude) significantly influences analgesic outcomes. The optimal frequency (1.0 Hz) and amplitude (1.0–1.5 mm) for acupunctural analgesia were identified in AA rat models. This parameter-dependent therapeutic effect is mediated by mast cell degranulation and the release of chemicals, particularly histamine, and is likely associated with local mechanical interactions between the needle and tissue.

## Data Availability

The original contributions presented in the study are included in the article/Supplementary Material, further inquiries can be directed to the corresponding authors.
