# Supplementary material for: Influence of acupuncture intensity on analgesic effects in AA rat models
Source: Front Bioeng Biotechnol. 2024 Dec 11;12:1502535. doi: 10.3389/fbioe.2024.1502535 (PMC11668573; doi:10.3389/fbioe.2024.1502535)
Supplement: Supplementary file 1 [file DataSheet1.docx]

Supporting materials on

‘Influence of acupuncture intensity on analgesic effects in AA rat models’

Yi-Xuan Wang^1,2^, Yu-Hang Liu^1,2^, Zi-Liang Zhang^1,2^, Xuan Qiao^1,2^, Ying-Chen Li^1,2^,

Liu-Jie Ren^3^, Guang-Hong Ding^1,2^, Wei Yao^1,2,*^, Yi Yu^4,*^

1. Department of Aeronautics and Astronautics, Fudan University, Shanghai 200433, China
2. Shanghai Key Laboratory of Acupuncture Mechanism and Acupoint Function, Shanghai 200433, China
3. Eye and ENT Hospital of Fudan University, Shanghai 200031, China
4. College of Medical Instruments, Shanghai University of Medicine & Health Sciences, Shanghai 201318, China

* Correspondence authors:

Wei Yao, PhD, Email: weiyao@fudan.edu.cn

Yi Yu, PhD, Email: 2200009@sumhs.edu.cn


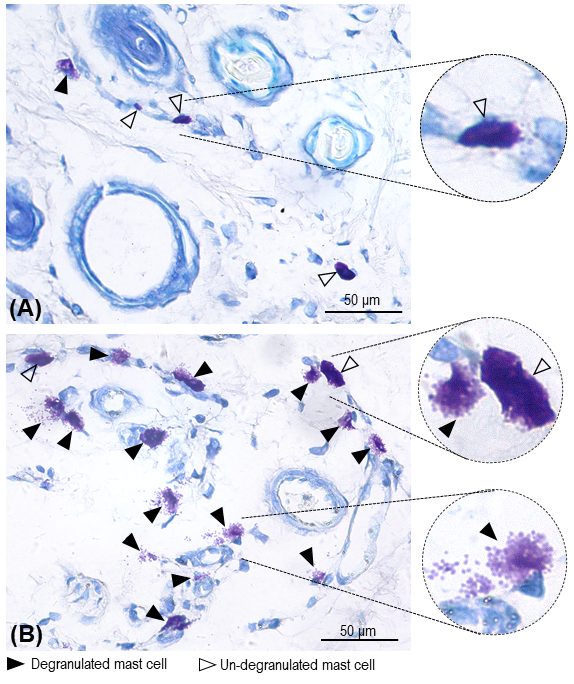


**Figure S1**. Example sections of stained mast cells within the acupoint tissues. The mast cells are stained purple and can be classified into two states: degranulated and un-degranulated. Un-degranulated mast cells exhibit relatively clear cell borders, whereas degranulated mast cells are characterized by numerous small granules distributed around the cell body. (A) demonstrates tissue from an untreated acupoint, only 1 out of 4 mast cells (25.0%) is degranulated. (B) demonstrates tissue from a treated acupoint, in which 14 out of 16 (87.5%) mast cells are degranulated.


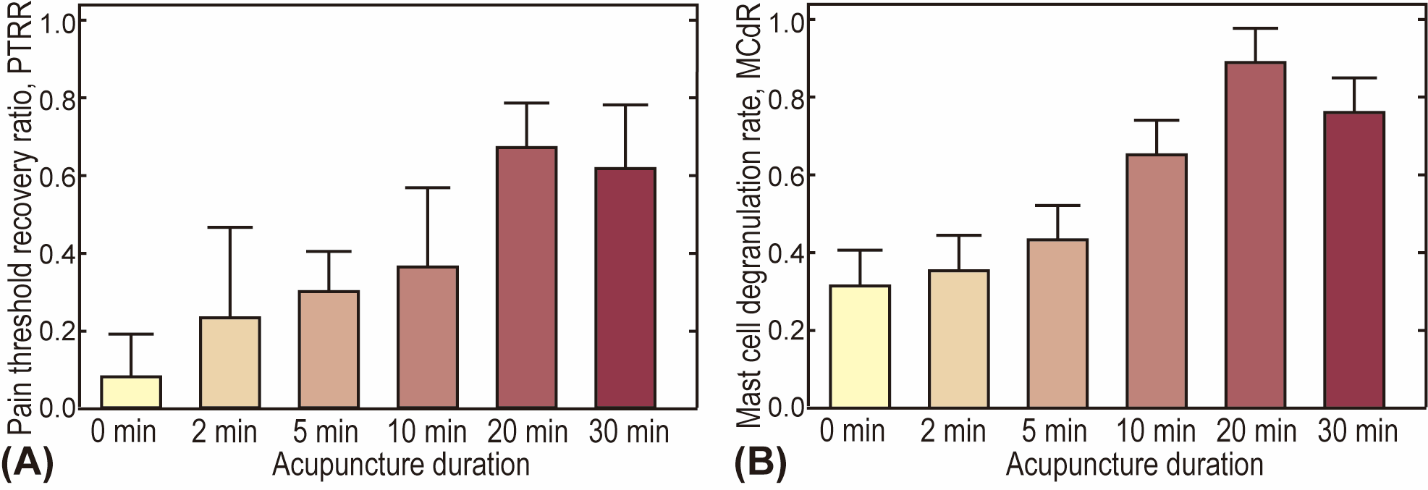


**Figure S2**. Influence of acupuncture duration on PTRR and MCdR. The stimulus parameters: frequency 2 Hz, and amplitude 1.5 mm. Paper based on this data has already been accepted by Journal of Fudan University (Natural Science), titled “correlation analysis between acupuncture time and analgesic effects (in Chinese)”. The maximum PTRR reaches above 0.6 after 20 minutes, correlated with a maximum MCdR of mostly above 80%.

**Table S1.** Original data of this study (N=78 rats)

| **Group** | **Subgroup** | **Animal** | **Thermal pain threshold, PWL, seconds** | | | | | **PTRR** | **MCdR** |
| --- | --- | --- | --- | --- | --- | --- | --- | --- | --- |
|  |  |  | Acclimation  Day -2 | Acclimation  Day -1 | Baseline $L_{0}$ | Model $L_{1}$  Day 2 | Treatment $L_{2}$, Day 2 |  |  |
| **RA** | **Subgroup 1**  0.5 Hz  1.0 mm | #1 | 10.78 | 13.07 | 11.93 | 5.07 | 6.63 | 0.23 | 0.58 |
|  |  | #2 | 13.02 | 11.33 | 12.18 | 4.73 | 6.05 | 0.18 | 0.50 |
|  |  | #3 | 11.12 | 16.93 | 14.03 | 5.48 | 5.80 | 0.04 | 0.55 |
|  |  | #4 | 16.60 | 12.47 | 14.54 | 3.68 | 8.92 | 0.48 | 0.55 |
|  |  | #5 | 13.72 | 11.16 | 12.44 | 3.08 | 9.23 | 0.66 | 0.57 |
|  |  | #6 | 15.35 | 15.42 | 15.39 | 2.63 | 9.83 | 0.56 | 0.58 |
|  |  | **Mean** | **13.43** | **13.40** | **13.41** | **4.11** | **7.74** | **0.36** | **0.56** |
|  |  | **S.D.** | **2.10** | **2.11** | **1.30** | **1.05** | **1.62** | **0.22** | **0.03** |
|  | **Subgroup 2**  1.0 Hz  1.0 mm | #1 | 15.11 | 13.56 | 14.34 | 4.25 | 9.32 | 0.50 | 0.65 |
|  |  | #2 | 10.60 | 12.03 | 11.32 | 5.02 | 8.28 | 0.52 | 0.65 |
|  |  | #3 | 11.03 | 10.88 | 10.96 | 5.69 | 9.84 | 0.79 | 0.82 |
|  |  | #4 | 14.26 | 18.45 | 16.36 | 3.91 | 8.95 | 0.40 | 0.65 |
|  |  | #5 | 12.84 | 14.41 | 13.63 | 3.36 | 7.30 | 0.38 | 0.65 |
|  |  | #6 | 15.00 | 12.35 | 13.68 | 4.15 | 9.12 | 0.52 | 0.83 |
|  |  | **Mean** | **13.14** | **13.61** | **13.38** | **4.40** | **8.80** | **0.52** | **0.71** |
|  |  | **S.D.** | **1.81** | **2.44** | **1.83** | **0.76** | **0.82** | **0.13** | **0.08** |
|  | **Subgroup 3**  2.0 Hz  1.0 mm | #1 | 11.23 | 14.77 | 13.00 | 4.81 | 7.36 | 0.31 | 0.54 |
|  |  | #2 | 14.12 | 17.15 | 15.64 | 4.60 | 8.08 | 0.32 | 0.66 |
|  |  | #3 | 9.33 | 10.07 | 9.70 | 2.50 | 5.93 | 0.48 | 0.60 |
|  |  | #4 | 10.85 | 12.21 | 11.53 | 2.98 | 6.99 | 0.47 | 0.48 |
|  |  | #5 | 9.57 | 12.45 | 11.01 | 1.91 | 5.51 | 0.40 | 0.56 |
|  |  | #6 | 11.75 | 15.06 | 13.41 | 3.47 | 8.36 | 0.49 | 0.71 |
|  |  | **Mean** | **11.14** | **13.62** | **12.38** | **3.38** | **7.04** | **0.41** | **0.59** |
|  |  | **S.D.** | **1.59** | **2.30** | **1.91** | **1.05** | **1.04** | **0.07** | **0.08** |
|  | **Subgroup 4**  3.0 Hz  1.0 mm | #1 | 12.42 | 12.31 | 12.37 | 3.62 | 4.63 | 0.12 | 0.50 |
|  |  | #2 | 12.23 | 11.51 | 11.87 | 2.81 | 3.31 | 0.06 | 0.44 |
|  |  | #3 | 13.61 | 10.10 | 11.86 | 3.52 | 4.71 | 0.14 | 0.48 |
|  |  | #4 | 13.79 | 14.49 | 14.14 | 4.13 | 5.16 | 0.10 | 0.52 |
|  |  | #5 | 12.67 | 17.62 | 15.15 | 3.53 | 5.57 | 0.18 | 0.42 |
|  |  | #6 | 14.72 | 16.72 | 15.72 | 4.90 | 6.37 | 0.14 | 0.56 |
|  |  | **Mean** | **13.24** | **13.79** | **13.52** | **3.75** | **4.96** | **0.12** | **0.49** |
|  |  | **S.D.** | **0.88** | **2.73** | **1.56** | **0.64** | **0.94** | **0.04** | **0.05** |
|  | **Subgroup 5**  4.0 Hz  1.0 mm | #1 | 10.66 | 14.46 | 12.56 | 4.31 | 4.67 | 0.04 | 0.33 |
|  |  | #2 | 9.38 | 14.91 | 12.15 | 4.85 | 6.66 | 0.25 | 0.36 |
|  |  | #3 | 15.03 | 13.06 | 14.05 | 3.17 | 5.46 | 0.21 | 0.50 |
|  |  | #4 | 12.20 | 17.30 | 14.75 | 4.16 | 5.83 | 0.16 | 0.50 |
|  |  | #5 | 17.04 | 18.97 | 18.01 | 4.61 | 4.93 | 0.02 | 0.26 |
|  |  | #6 | 17.14 | 14.59 | 15.87 | 4.31 | 5.16 | 0.07 | 0.49 |
|  |  | **Mean** | **13.58** | **15.55** | **14.56** | **4.24** | **5.45** | **0.13** | **0.41** |
|  |  | **S.D.** | **3.02** | **1.98** | **1.99** | **0.53** | **0.65** | **0.08** | **0.10** |
|  | **Subgroup 6**  2.0 Hz  0.5 mm | #1 | 14.54 | 11.07 | 12.81 | 2.73 | 3.69 | 0.10 | 0.52 |
|  |  | #2 | 10.59 | 10.20 | 10.40 | 3.25 | 4.96 | 0.24 | 0.37 |
|  |  | #3 | 8.75 | 9.23 | 8.99 | 5.33 | 5.80 | 0.13 | 0.18 |
|  |  | #4 | 13.31 | 12.88 | 13.10 | 3.77 | 5.31 | 0.17 | 0.27 |
|  |  | #5 | 9.20 | 14.07 | 11.64 | 4.08 | 3.78 | -0.04 | 0.38 |
|  |  | #6 | 8.49 | 11.92 | 10.21 | 2.85 | 3.04 | 0.03 | 0.51 |
|  |  | **Mean** | **10.81** | **11.56** | **11.19** | **3.67** | **4.43** | **0.10** | **0.37** |
|  |  | **S.D.** | **2.32** | **1.62** | **1.47** | **0.88** | **0.99** | **0.09** | **0.12** |
|  | **Subgroup 7**  2.0 Hz  1.5 mm | #1 | 12.76 | 14.21 | 13.49 | 3.96 | 6.28 | 0.24 | 0.55 |
|  |  | #2 | 13.19 | 12.53 | 12.86 | 4.71 | 8.34 | 0.45 | 0.58 |
|  |  | #3 | 12.29 | 13.19 | 12.74 | 3.63 | 6.80 | 0.35 | 0.58 |
|  |  | #4 | 15.77 | 13.79 | 14.78 | 3.67 | 6.37 | 0.24 | 0.54 |
|  |  | #5 | 16.63 | 13.55 | 15.09 | 2.92 | 6.10 | 0.26 | 0.42 |
|  |  | #6 | 10.79 | 11.95 | 11.37 | 3.36 | 5.66 | 0.29 | 0.54 |
|  |  | **Mean** | **13.57** | **13.20** | **13.39** | **3.71** | **6.59** | **0.30** | **0.54** |
|  |  | **S.D.** | **2.02** | **0.76** | **1.27** | **0.55** | **0.85** | **0.07** | **0.06** |
|  | **Subgroup 8**  2.0 Hz  2.0 mm | #1 | 5.29 | 11.82 | 8.56 | 5.01 | 4.81 | -0.06 | 0.17 |
|  |  | #2 | 11.10 | 12.05 | 11.58 | 3.25 | 5.29 | 0.25 | 0.28 |
|  |  | #3 | 9.52 | 10.14 | 9.83 | 2.16 | 4.35 | 0.29 | 0.26 |
|  |  | #4 | 12.01 | 11.30 | 11.66 | 3.51 | 3.62 | 0.01 | 0.49 |
|  |  | #5 | 11.38 | 10.36 | 10.87 | 4.06 | 4.85 | 0.12 | 0.51 |
|  |  | #6 | 11.89 | 15.58 | 13.74 | 3.96 | 6.29 | 0.24 | 0.47 |
|  |  | **Mean** | **10.20** | **11.88** | **11.04** | **3.66** | **4.87** | **0.14** | **0.36** |
|  |  | **S.D.** | **2.34** | **1.80** | **1.61** | **0.87** | **0.82** | **0.13** | **0.13** |

**Table S1 (Continued).** Original data of this study (N=78 rats)

| **Group** | **Subgroup** | **Animal** | **Thermal pain threshold, PWL, seconds** | | | | | **PTRR** | **MCdR** |
| --- | --- | --- | --- | --- | --- | --- | --- | --- | --- |
|  |  |  | Acclimation  Day -2 | Acclimation  Day -1 | Baseline $L_{0}$ | Model $L_{1}$  Day 2 | Treatment $L_{2}$, Day 2 |  |  |
| **MA** | ~2.0 Hz  ~1.0 mm | #1 | 11.66 | 12.99 | 12.33 | 3.62 | 7.26 | 0.42 | 0.73 |
|  |  | #2 | 13.34 | 13.06 | 13.20 | 4.33 | 6.89 | 0.29 | 0.68 |
|  |  | #3 | 12.24 | 13.50 | 12.87 | 2.89 | 7.11 | 0.42 | 0.75 |
|  |  | #4 | 16.84 | 17.37 | 17.11 | 3.28 | 7.09 | 0.28 | 0.62 |
|  |  | #5 | 14.45 | 17.42 | 15.94 | 3.77 | 6.39 | 0.22 | 0.65 |
|  |  | #6 | 11.22 | 11.49 | 11.36 | 2.70 | 7.05 | 0.50 | 0.56 |
|  |  | **Mean** | **13.29** | **14.31** | **13.80** | **3.43** | **6.97** | **0.35** | **0.67** |
|  |  | **S.D.** | **1.91** | **2.27** | **2.04** | **0.55** | **0.28** | **0.10** | **0.06** |
| **Model** | **-** | #1 | 9.31 | 11.49 | 10.40 | 2.94 | 3.89 | 0.13 | 0.26 |
|  |  | #2 | 11.51 | 19.40 | 15.46 | 2.90 | 4.19 | 0.10 | 0.26 |
|  |  | #3 | 15.63 | 11.96 | 13.80 | 3.69 | 4.70 | 0.10 | 0.32 |
|  |  | #4 | 9.66 | 9.12 | 9.39 | 3.77 | 3.87 | 0.02 | 0.20 |
|  |  | #5 | 12.46 | 10.75 | 11.61 | 4.29 | 3.93 | -0.05 | 0.25 |
|  |  | #6 | 11.27 | 15.27 | 13.27 | 4.72 | 5.12 | 0.05 | 0.32 |
|  |  | **Mean** | **11.64** | **13.00** | **12.32** | **3.72** | **4.28** | **0.06** | **0.27** |
|  |  | **S.D.** | **2.09** | **3.41** | **2.07** | **0.66** | **0.47** | **0.06** | **0.04** |
| **NR** | **-** | #1 | 14.75 | 13.38 | 14.07 | 2.97 | 2.51 | -0.04 | 0.31 |
|  |  | #2 | 14.21 | 15.04 | 14.63 | 4.03 | 6.09 | 0.19 | 0.27 |
|  |  | #3 | 13.97 | 17.26 | 15.62 | 5.12 | 6.24 | 0.11 | 0.19 |
|  |  | #4 | 11.40 | 8.52 | 9.96 | 5.13 | 5.64 | 0.11 | 0.23 |
|  |  | #5 | 10.89 | 11.64 | 11.27 | 5.90 | 5.50 | -0.07 | 0.48 |
|  |  | #6 | 13.18 | 10.44 | 11.81 | 3.97 | 5.77 | 0.23 | 0.54 |
|  |  | **Mean** | **13.07** | **12.71** | **12.89** | **4.52** | **5.29** | **0.09** | **0.34** |
|  |  | **S.D.** | **1.44** | **2.90** | **2.01** | **0.96** | **1.27** | **0.11** | **0.13** |
| **Cle**  **+**  **Acu** | 1.0 Hz  1.0 mm | #1 | 10.49 | 14.10 | 12.30 | 5.04 | 4.73 | -0.04 | 0.75 |
|  |  | #2 | 14.61 | 12.98 | 13.79 | 4.70 | 5.56 | 0.09 | 0.47 |
|  |  | #3 | 15.94 | 13.49 | 14.72 | 6.34 | 5.90 | -0.05 | 0.50 |
|  |  | #4 | 15.54 | 15.11 | 15.33 | 4.29 | 5.27 | 0.09 | 0.65 |
|  |  | #5 | 17.64 | 11.80 | 14.72 | 5.73 | 5.11 | -0.07 | 0.86 |
|  |  | #6 | 9.53 | 16.61 | 13.07 | 5.87 | 5.97 | 0.01 | 0.72 |
|  |  | **Mean** | **13.96** | **14.01** | **13.99** | **5.33** | **5.42** | **0.01** | **0.66** |
|  |  | **S.D.** | **2.95** | **1.54** | **1.05** | **0.71** | **0.44** | **0.07** | **0.14** |
| **Saline**  **+**  **Acu** | 1.0 Hz  1.0 mm | #1 | 12.99 | 11.69 | 12.34 | 5.91 | 10.17 | 0.66 | 0.92 |
|  |  | #2 | 13.86 | 15.57 | 14.72 | 5.70 | 9.35 | 0.40 | 0.56 |
|  |  | #3 | 13.21 | 11.68 | 12.45 | 4.09 | 7.33 | 0.39 | 0.91 |
|  |  | #4 | 13.89 | 14.84 | 14.37 | 4.73 | 9.34 | 0.48 | 0.88 |
|  |  | #5 | 11.14 | 11.19 | 11.17 | 3.19 | 8.29 | 0.64 | 0.76 |
|  |  | #6 | 15.04 | 12.24 | 13.64 | 3.79 | 10.86 | 0.72 | 0.51 |
|  |  | **Mean** | **13.36** | **12.87** | **13.11** | **4.57** | **9.22** | **0.55** | **0.76** |
|  |  | **S.D.** | **1.19** | **1.69** | **1.24** | **0.99** | **1.16** | **0.13** | **0.16** |
